# In Vitro Antilipidic and Antithrombotic Activities of *Plectranthus glandulosus* (Lamiaceae) Leaves Extracts and Fractions

**DOI:** 10.1155/2022/4145659

**Published:** 2022-02-07

**Authors:** Djamila Zouheira, Sylvie Lea Wansi Ngnokam, Sylviane Laure Poualeu Kamani, Billy Toussie Tchegnitegni, Jean Bosco Jouda, Jean Romuald Mba, Moïse Legentil Nchouwet, Njini Gael Nfor, Alain K. Nyirimigabo, Théodora Kopa Kowa, Gabriel A. Agbor

**Affiliations:** ^1^Department of Animal Biology, Faculty of Sciences, University of Dschang, Cameroon; ^2^Centre for Research on Medicinal Plants and Traditional Medicine (CRPMT), Institute of Medical Research and Medicinal Plants Studies (IMPM), Yaoundé, Cameroon; ^3^Department of Chemistry, Faculty of Science, University of Dschang, P. O. Box 67, Dschang, Cameroon; ^4^Department of Chemical Engineering, School of Chemical Engineering and Mineral Industries, University of Ngaoundere, P.O. Box 454 Ngaoundere, Cameroon; ^5^School of Medicine and Pharmacy, College of Medicine and Health Sciences, University of Rwanda, Rwanda; ^6^Rwanda Food and Drug Authority (RFDA), Ministry of Health (MOH), Kigali, Rwanda

## Abstract

**Objective:**

The present study investigated the effect of the leaves extracts and fractions of *Plectranthus glandulosus* on the inhibition of pancreatic lipase, cholesterol esterase, adipocytes lipid uptake, and antithrombotic activity which may be important in atherosclerosis development.

**Methods:**

Aqueous, ethanolic, and hydroethanolic extracts of *Plactranthus glandulosus* were prepared by maceration. The hydroethanolic extract was fractionated into *n*-hexane, ethylacetate, and *n-*butanol fractions and their inhibition of pancreatic lipase, cholesterol esterase, adipocytes lipid uptake, and antithrombotic activities measured. Liquid chromatography-high resolution mass spectrometry (LC-HRMS) analysis was carried out to determine phytochemical constituents present in the extracts.

**Results:**

The standard orlistat exhibited a higher inhibitory activity on pancreatic lipase and cholesterol esterase (16.31 *μ*g/mL and 15.75 *μ*g/mL, respectively) compared to ethyl acetate fraction (IC_50_, 17.70 *μ*g/mL and IC_50_, 24.8 *μ*g/mL, respectively). Among crude extract, hydroethanolic extract showed a better inhibition against pancreatic lipase (IC_50_, 21.06 *μ*g/mL) and cholesterol esterase (IC_50_, 25.14 *μ*g/mL) though not comparable to the effect of orlistat. The best lipid uptake inhibition was observed in the hydroethanolic extract (IC_50_, 45.42 *μ*g/mL) followed by the ethyl acetate fraction (IC_50_, 47.77 *μ*g/mL). A better antithrombolytic activity was exhibited by the ethyl acetate fraction at all concentrations (50-800 *μ*/mL), while hydroethanolic extract exhibited the best activity among crude extract. However, these were not comparable to the standard aspirin. The LC-HRMS analysis revealed the presence of 7-*O*-methyl luteolin 5-*O*-*β*-D-glucopyranoside, chrysoeriol 5-*O*-*β*-D-glucopyranoside, 5,7-dihydroxy-3,2′,4′-trimethoxyflavone, and plectranmicin as major compounds in both hydroethanolic extract and ethyl acetate fraction.

**Conclusion:**

Thus, our finding supports the traditional use of this plant, which might provide a potential source for future antiatherosclerotic drug discovery.

## 1. Introduction

Atherosclerosis is the major cause of stroke and heart attack, which are the leading cause of morbidity and mortality worldwide [1]. The prevalence is expected to be 23.6 million deaths by the year 2030 from cardiovascular diseases in the world, mainly from stroke and heart attacks, with the greatest increases expected in low- and middle-income developing countries [2]. The dysregulation of lipid metabolism and aberrant inflammatory responses in the arterial walls at predisposed sites plays a central role from initiation to the progression and eventually rupture of the atherosclerotic plaque [3, 4] which is associated with thrombotic vessel occlusion, the main cause of atherosclerosis complications [5].

Hyperlipidemia is the most important risk factor of atherosclerosis initiation [6]. Hypercholesterolemia is increasing day by day due to the ingestion of high fat diet. In the digestive system, fats need to be broken down before they can be absorbed. Among fats from the diet, triglycerides and cholesterol are the most important because they are involved in increasing level of atherogenic low-density lipoprotein (LDL). Triglycerides and cholesterol are hydrolyzed by specific enzymes which facilitate their digestion and absorption. Triglycerides are subjected to the action of pancreatic lipases, which hydrolyze triglycerides into monoacylglycerols and free fatty acids [7]. Dietary cholesterol consists of both free and esterified cholesterol. The hydrolysis of cholesterol esters in the lumen of the small intestine is catalyzed by pancreatic cholesterol esterase, which liberates free cholesterol. Excess release of fatty acids in the plasma will lead to very low-density lipoprotein (VLDL) production by the liver which may be transformed by lipoprotein lipase into intermediate density lipoprotein (IDl) and then converted into smaller atherogenic LDL [8]. High plasma cholesterol stimulates an increase in the secretion of plasma LDL the main carrier of cholesterol in plasma [9]. Hyperlipidemia may also increase endothelial dysfunction and oxidative stress, which can lead to endothelial damage [10], promoting the infiltration of LDL cholesterol in the arterial wall, their oxidation by reactive oxygen species, inflammation, and foam cell formation in the arterial intima, which is the initial step of atherosclerosis formation.

On the other hand, hyperlipidemia is involved in adipogenesis, which is characterized by the enlargement of adipocytes due to impaired adipocytes' differentiation and infiltration of macrophages into adipose tissue that lead to a chronic state of inflammation in the adipocytes [11]. The inflammation state is associated with a reduction in the secretion of adiponectin, which leads to a decrease in the level of anti-atherogenic high density lipoprotein (HDL) and increases triglycerides [12]. Chronic state of inflammation may also lead to increased secretion of proinflammatory cytokines such as interleukine (IL-6, IL-8), tumor necrosis factor alpha (TNF-*α*), interferon-*γ* (INF- *γ*), and monocyte chemoattractant protein 1 (MCP-1) which are involved in endothelial dysfunction [11, 13]. After the formation of atherosclerotic plaque, the most clinical manifestation involves precipitating thrombin that obstruct blood flow to the heart (coronary heart disease), brain (ischemic stroke), or lower extremities (peripheral vascular disease) [5].

Following the pathophysiology of atherosclerosis, a wide range of drugs from antihyperlipidemic to antithrombotic are suggested for the treatment of atherosclerosis. Nonetheless, some of these drugs have been marred by numerous side effects such as myalgia, arthralgia, elevated liver enzymes, elevated blood glucose, dyspepsia and constipation, severe contraindications, and drug interactions (statins and fibrates) [14]. Orlistat, a gastric and pancreatic lipase inhibitor, is currently approved for long-term treatment, but it has gastrointestinal side effects such as bloating, oily spotting, steatorrhea, fecal urgency, and fecal incontinence [15, 16]. Digestive hemorrhage, cerebral meningeal hemorrhage, and intravisceral hematoma are observed with thrombolytic drugs [17]. In addition, it must be mentioned that these drugs have exuberant costs that a common man may not be able to afford for long-term treatment. Obviously, this has led to the search of novel lead molecules, preferably from natural products possessing antilipidemic and antithrombotic properties with lesser or no side effects and that are affordable to a large spectrum of population compared to the existing antilipidemic and antithrombotic drugs. Interestingly, a large number of earlier studies have reported many plant secondary metabolites, especially phenolic compounds, and have good inhibitory effects toward digestive enzymes such as pancreatic lipase and cholesterol esterase enzymes [7, 18–20]. Dietary polyphenols may also suppress the growth of adipose tissue through their antiangiogenic activity and by modulating adipocyte metabolism [21], as well as thrombolytic activity by dissolving blood clots [22]. Moreover, alkaloids, tannins, and saponins are involved in thrombolytic activity [23].

*Plecthrantus glandulosus (P. glandulosus)* Hook. F. (Lamiaceae) is a climbing herbaceous plant widely distributed in West, Central, and South of Africa [24]*. P. glandulosus* leaves are traditionally used in the Adamaoua Region of Cameroon for the treatment of obesity, hypertension, stroke, and heart attack, though no scientific investigation regarding these claims is available. However, earlier scientific data reported its implication in the treatment of influenza, cough, chest complaints, and as food preservatives [25]. The antinociceptive, anti-inflammatory, and antioxidant activities of the plant [26–29] as well as the inhibition of LDL oxidation have been reported [29]. Phytochemical examination of *P. glandulosus* revealed the presence of secondary metabolites such as alkaloids, flavonoids, saponins, terpenoids, steroids, tannins, and glucosides [28–31]. Further phytochemical studies isolated a methoxylated flavonoid derivative plectranmicin, a monoterpene derivative plectranmicinol, and seven known compounds (5-hydroxy-3,7,2′,4′-tetramethoxyflavone, 5,7-dihydroxy-3,2′,4′-trimethoxyflavone, 7-hydroxy-5,6,4′-trimethoxyflavonen, 3-epi-betulinic acid, 3*-O-β*-D-glucopyranosyl stigmasterol, *β*-sitosterol, and 4-epi-fridelin) [32]. Nanmeni et al. [33] isolated seventeen compounds form de ethanolic whole plant extract including the mixture of stigmasterol and *β*-sitosterol, oleanolic acid, pilloin, *β*-sitosterol 3-*O*-*β*-D-glucopyranoside, chrysoeriol, luteolin 7-methyl ether, 5-hydroxy-7,4′-dimethoxyflavone, mixture of maslinic acid and benthamic acid, 5,6-dihydroxy-7,3′,4′-trimethoxyflavone, ladanein, hederagenin, cylicodiscic acid, mixture of chrysoeriol 5-*O*-*β*-D-glucopyranoside and 7-*O*-methyl luteolin 5-*O*-*β*-D-glucopyranoside, and galuteolin. Tedonkeu et al. [34] isolated a new nor-triterpenoid along with seventeen known compounds including seven triterpenoids, nine flavonoids, and one steroid from the ethanol extract of the whole plant.

In the present study, we evaluated the effect *P. glandulosus* leaves extracts and fractions on the inhibition of pancreatic lipase, cholesterol esterase, adipocyte lipid uptake, and thrombolytic activity. A phytochemical screening was carried out to confirm the presence of already reported bioactive components and to isolate new ones if identified.

## 2. Materials and Methods

### 2.1. Chemicals

Porcine pancreatic lipase, phosphate buffer (PBS, pH 7.4), *p*-nitrophenyl butyrate, dimethylformamide, taurocholic acid, porcine pancreatic cholesterol esterase, orlistat, penicillin, streptomycin, bovine serum albumin (BSA), oleic acid, Oil Red-O, isopropanol, formalin, aspirin, sodium phosphate monobasic, and sodium phosphate dibasic dihydrate were purchased from Sigma-Aldrich, Co. (St. Louis, MO, USA).

### 2.2. Plant Material

Fresh leaves of *P. glandulosus* were collected in the month of January 2017 between 7 : 30 am and 9 : 00 pm (Cameroon time) in Ngaoundere, Adamawa Region of Cameroon. The plant species identification was confirmed at the National Herbarium of Yaoundé Cameroon (Voucher specimen n°41168-HCN) [28].

### 2.3. Crude Extract Preparation

*Plectranthus glandulosus (P. glandulosus)* crude extracts were prepared as previously described [28]. In brief, aqueous, ethanolic, and hydroethanolic extracts were prepared by maceration at room temperature for 48 h as follows: aqueous extract was obtained by maceration of 1200 g of the powdered material in 10 L of distilled, ethanolic extract by maceration of 1200 g of powder in 14 L of ethanol, and hydroethanolic extract by maceration of 2400 g into ethanol/water (70 : 30 v/v). After filtration, the solvents were evaporated using a rotary evaporator (BUCHI), and the three extracts were dried separately in a ventilated drying oven (MANESTY- PETRIE) at 50°C for 24 hours. The extraction yield was 11.15% for aqueous extract, 2.88% for ethanolic extract, and 10.96% for hydroethanolic extract, respectively.

### 2.4. Fractionation of Plant Materials

The fractionation of *P. glandulosus* leaves was done as describe in previous report [28]. The hydroethanolic crude extract (190 g) was fractioned with *n-*hexane, ethyl acetate, and *n*-butanol in increasing order of polarity. At the end of the fractionation, *n-*hexane fraction, ethyl acetate fraction, *n*-butanol fraction, and residual fraction were obtained after evaporation of the solvents (rotary evaporator BUCHI) and dried (oven MANESTY- PETRIE) at 50°C for 24 hours. The fractionation yields were 34.11% for *n-*hexane fraction, 10.34% for ethyl acetate fraction, 3.15% for *n*-butanol, and 42.26% for residual fraction.

### 2.5. Pancreatic Lipase Inhibition Assay

Pancreatic lipase inhibitory activity of leaves extracts and fractions of *P. glandulosus* were carried out according to the method described by Kim et al. [35] with some modifications. The reaction mixture consisted of 80 *μ*L of each extract/fraction at different concentrations (12.5, 25, 50, 100, 200 *μ*g/mL), 20 *μ*L of porcine pancreatic lipase (4 mg/mL), and 90 *μ*L of phosphate buffer (PBS, pH 7.4). After 15 min of incubation at 37°C, the reaction was started by adding 10 *μ*L of *p*-nitrophenyl butyrate (10 mM) in dimethylformamide and was allowed to proceed at 37°C for 30 min. Lipase inhibitory activity of leaves extracts and fractions was determined by measuring the hydrolysis of *p*-NPB to *p*-nitrophenol (yellow color) at 405 nm using a microplate reader. Orlistat was used as the positive control. Inhibition of lipase activity was expressed as the percentage decrease in optical density when pancreatic lipase was incubated with leaves extracts and fractions. Lipase inhibition (%) was calculated according to the following formula:

(1) $$\begin{array}{c} \text{Inhibition percentage}=\left[\frac{\left(\text{Optical density of blank}-\text{Optical density test}\right)}{\text{Optical density blank}}\right]\text{ x }100. \end{array}$$

Antilipase activity was given as IC_50_ values (the concentrations of leaves extracts, fractions, and orlistat that inhibited the hydrolysis of *p*-NPB to *p*-nitrophenol by 50%, *n* = 3).

### 2.6. Cholesterol Esterase Inhibition Assay

The pancreatic cholesterol esterase inhibition activity of leaves extracts and fractions of *P. glandulosus* were performed spectrophotometrically based on the method of Adisakwattana et al. [36]. The extract/fraction (50 *μ*L) at different concentrations (12,5, 25, 50, 100, 200 *μ*g/mL) was incubated with mixtures containing 50 *μ*L of taurocholic acid (5.16 mM), 50 *μ*L of *p*-nitrophenyl butyrate substrate (0.2 mM), and 90 *μ*L of sodium phosphate buffer (100 mM, pH 7.0). The reaction was initiated by adding 50 *μ*l of porcine pancreatic cholesterol esterase (1 *μ*g/mL). After incubation for 5 min at 25°C, the absorbance was measured at 405 nm. Orlistat was used as the positive control. The percentage inhibition was calculated as

(2) $$\begin{array}{c} \text{Inhibition percentage}=\left[\frac{\left(\text{Optical density of blank}-\text{Optical density test}\right)}{\text{Optical density blank}}\right]\text{ x }100. \end{array}$$

Anticholesterol esterase activity was given as IC_50_ values (the concentrations of leaves extracts, fractions, and orlistat that inhibited the hydrolysis of *p*-NPB to *p*-nitrophenol by 50%, *n* = 3).

### 2.7. Lipid Accumulation Inhibition Assay

#### 2.7.1. SW872 Cell Line

The human liposarcoma cell line, SW872, was obtained from American Type Culture Collection (ATCC). These cells have a physiological response, similar to mature adipocytes. Compared to 3 T3-L1 which are mouse adipocytes, these cells have the advantage of being human origin.

#### 2.7.2. SW872 Cell Line Culture

Cells were cultured in DMEM/Ham's F-12 containing 10% FBS (Atlas Biologicals, Fort Collins, CO) and 100 U/mL penicillin/in streptomycin 5% CO_2_/95% air at 37°C. The culture medium was replaced every 2 or 3 days, until the confluence is reached at 100%.

#### 2.7.3. Evaluation of the Effects of *P. glandulosus* Leaves Extracts and Fractions on SW-872 Differentiation

The effects of leaves extracts and fractions of *P. glandulosus* on the differentiation of SW-872 preadipocytes into mature adipocytes were assessed in the inhibition of lipid uptake according to Dordevic et al. [37] method. Cells were incubated in DMEM/Ham's F-12 containing 1% BSA and 0.6 mol/l oleic acid (differentiating agent). Leaves extracts and fractions at different concentrations (25, 50, 100, and 200 *μ*g/mL) were added to the wells corresponding to the test wells. We considered undifferentiated cells as negative control and SW-872 cells treated only with oleic acid as a positive control. Cells were cultured for 3 days until 90% of confluences were reached.

After cells were stained with oil red O according to Ramírez-Zacarías et al. [38] method, with slight modifications, for that, a stock solution of Oil Red-O, 5% was prepared in isopropanol. The solution was filtered (0.2 *μ*m) and stored at 4°C. The working solution was prepared by diluting the stock solution with distilled water (6 : 4), followed by incubation at room temperature for 20 min, and the solution was further filtered. Cells were washed twice with phosphate buffer (PBS, pH 7.4) and fixed with formaline (10%). Next, the fixed cells were washed three times with deionized water and dried. Oil Red-O solution (1.5 mL/well) was then added in the plate wells. Stained cells were incubated at room temperature for 30 min, and photos were taken using an electronic microscope (inverted microscope) associated with a digital camera (×200 magnification) to observe the lipid droplets accumulated in SW-872 cells. After these, isopropanol was added to the wells to allow lipid hydrolysis, the supernatant was collected, and the optical density was measured at 492 nm. Lipid uptake inhibition percentage was calculated [39] according to the following formula:

(3) $$\begin{array}{c} \text{Inhibition}=\left[\frac{\left(\text{Optical density of positive control}-\text{Optical density test}\right)}{\text{Optical density positive control}}\right]\text{ x }100. \end{array}$$

IC_50_ values were calculated (the concentrations of leaves extracts and fraction that inhibited the lipid uptake by 50%, *n* = 3).

### 2.8. Thrombolytic Assay

Blood sample used in this test was drawn by capillary tube used to disrupt the retrobulbar venous sinus located behind the eye (retroorbital bleeding) from healthy rats under pentobarbital sodium anesthesia (humane pharmaceutical grade, 78 mg/kg of body weight administered intraperitonially). The pain level of the animal was determined by no withdrawal of limb with pinching and by no response to a penlight shone in the eye. This technique was carried out in accordance with the practice and principles of the Institute of Medical Research and Medicinal Plants Studies, Yaoundé-Cameroon on the use of experimental animals respecting the 2011 Guide for the Care and Use of Laboratory Animals, 8th Edition and the Animal Welfare Act. These rats were provided by the animal house of the Institute of Medical Research and Medicinal Plants Studies (IMPM), Yaoundé, Cameroon.

The antithrombotic assay was realized according to Sweta et al. [40]. method. Briefly, 500 *μ*L of blood was transferred to each previously weighed test tube to form clots, and this was followed by incubation at 37°C for 45 minutes. After clot enhancement, serum was completely removed (aspirated around without disturbing the clot formed). Each tube having a clot was again weighed (clot weight = body weight of clot and tube-weight of tube alone). In every test tube containing clog, 100 *μ*L of leaves extract or fraction at different concentrations (50, 100, 200, 400, and 800 *μ*/mL) was added. All test tubes were then incubated at 37°C for 90 minutes and observed for clot lysis. After incubation, the fluid obtained was removed, and the tubes were again weighed. Weight difference before and after clot lysis was expressed in percentage. Aspirin and distilled water were used as positive and negative control, respectively. The test was carried out in triplicate for each concentration of leaves extract, fraction, and aspirin. The percentage of clot lysis was calculated according to the following formula:

(4) $$\begin{array}{c} \%\text{clot lysis}=\left[\frac{\text{Weight of the lysed clot}}{\text{Weight of clot before lysis}}\right]\text{ x }100. \end{array}$$

### 2.9. Quantification of Individual Phenolic Compounds in Aqueous, Hydroethanolic Extracts and Ethyl Acetate Fraction through LC-HRMS Analysis

Phenolic compounds contained in aqueous extract (extracts used by the traditional healers), hydroethanolic extract (most active crude extract), and ethyl acetate fraction (most active fraction) were quantified using analytical LC-MS. The analysis was performed on an Agilent 6220 ToF-MS with a Dual ESI-source, 1200 HPLC system with autosampler, degasser, binary pump, column oven, diode array detector, and a Hypersil Gold C18 column (1.9 *μ*m, 50 × 2.1 mm) with a gradient (in 11 min from 0% B to 98% B, back to 0% B in 0.5 min, total run time 15 min) at a flow rate of 300 *μ*L/min and column oven temperature of 40°C. HPLC solvent A consists of 94.9% water, 5% acetonitrile and 0.1% formic acid, solvent B of 5% water, 94.9% acetonitrile, and 0.1% formic acid. The mass spectra are recorded in both profile and centroid mode with the MassHunter Workstation Acquisition B.04.00 software (Agilent Technologies, Santa Clara, CA, USA). MassHunter Qualitative Analysis B.07.00 software (Agilent Technologies, Santa Clara, CA, USA) was used for processing and averaging of several single spectra.

### 2.10. Statistical Analysis

Data in the current study are presented as mean values (*n* = 3) ± standard deviation (SD). The statistical analysis was carried out using GraphPad Prism version 5.00 for windows (GraphPad software) in which one-way ANOVA was used and *p* < 0.05 was set as significant. IC_50_ values were calculated using nonlinear regression with GraphPad Prism 5.

## 3. Results

### 3.1. Effects of *P. glandulosus* Leaves Extracts and Fractions on Pancreatic Lipase Inhibition

**Figure 1:**
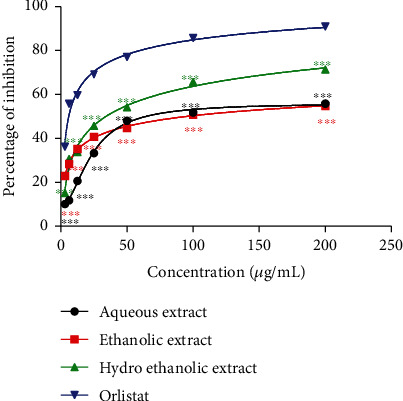
Effects of *P. glandulosus* leaves extracts on pancreatic lipase inhibition. Values are expressed as mean ± SD (*n* = 3); ^∗∗∗^*p* < 0.001 significant differences compared to orlistat.

**Figure 2:**
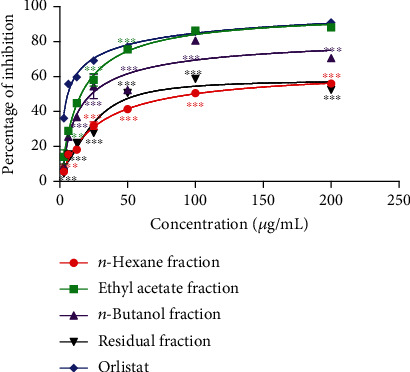
Effects of *P. glandulosus* leaves fractions on pancreatic lipase inhibition.

The inhibition of pancreatic lipase activity of different extracts and fractions of *P. glandulosus* leaves is presented in Figures 1 and 2. Orlistat the reference standard exhibited the best percentage inhibition of 90.97% at 200 *μ*g/mL which was comparable only to the ethyl acetate fraction percentage inhibition of 88.24%. Among extracts, the hydroethanolic extract showed the highest percentage inhibition of 71.33%, followed by ethanolic extract (54.27%) and lastly, aqueous extract (55.89%). Among fractions, ethyl acetate had the best percentage inhibition of 88.24% higher than that of *n*-butanol fraction (70.54%), residual fraction (57.42%), and *n-*hexane fraction (55.96%). All extracts and fractions presented a concentration response percentage inhibition activity with a sharp gradient up to 25 *μ*g/mL which then turns towards saturation at 50 *μ*g/mL.

The best IC_50_ for lipase inhibition was presented by orlistat (7.53 ± 0.52 *μ*g/mL) which is the reference drug showing its important inhibitory concentration. Among extracts and fractions, ethyl acetate fraction (24.20 ± 0.55 *μ*g/mL) presented the best IC_50_. This was closely followed by hydroethanolic extract (39.16 ± 0.17 *μ*g/mL), the n-butanol fraction (44.09 ± 1.41 *μ*g/mL), and the ethanolic extract (56.62 ± 2.12 *μ*g/mL). The residual fraction (71.15 ± 0.6 *μ*g/mL), aqueous extract (79.62 ± 1.26 *μ*g/mL), and *n-*hexane fraction (86.58 ± 0.07 *μ*g/mL) which presented high IC_50_ were the least effective (Table 1).

Values are expressed as mean ± SD (*n* = 3); ^∗∗∗^*p* < 0.001 significant differences compared to orlistat.

### 3.2. Effects of *P. glandulosus* Leaves Extracts and Fractions on Pancreatic Cholesterol Esterase Inhibition

**Figure 3:**
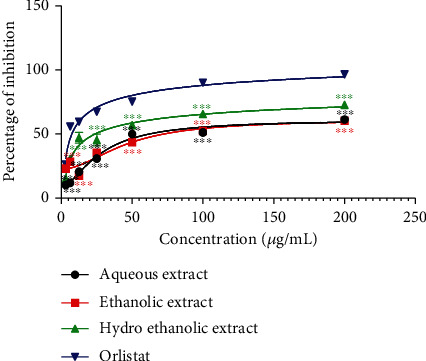
Effects of *P. glandulosus* leaves extracts on pancreatic cholesterol esterase inhibition. Values are expressed as mean ± SD (*n* = 3); ^∗∗∗^*p* < 0.001 significant differences compared to orlistat.

**Figure 4:**
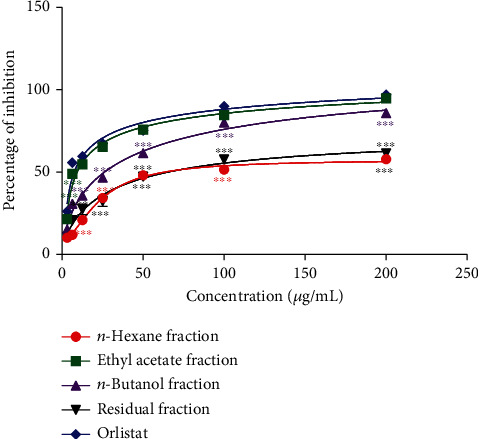
Effects of *P. glandulosus* leaves fractions on pancreatic cholesterol esterase inhibition. Values are expressed as mean ± SD (*n* = 3); ^∗∗∗^*p* < 0.001 significant differences compared to orlistat.

The effect of *P. glandulosus* leaves extracts and fractions on pancreatic cholesterol esterase inhibition is presented in Figures 3 and 4. *P. glandulosus* leaves extracts inhibited cholesterol esterase in concentration-dependent manner. However, this inhibition was significantly (*p* < 0.001) lower than that of orlistat at all concentrations (3.12 to 200 *μ*g/mL). The inhibition exhibited by hydroethanolic extract at the concentration 200 *μ*g/mL was 72.71%, followed by aqueous extract (61.34%) and ethanolic extract which exhibited inhibition percentage of 60.30%. The percentage inhibition showed by orlistat at this same concentration was 97%. Among fractions, the percentage inhibition activity of ethyl acetate was the highest and comparable to that of orlistat at a concentration range (12.5-200 *μ*g/mL). These inhibitions were significantly (*p* < 0.001) higher than that of *n-*hexane, *n*-butanol, and residual fractions. The percentage inhibition for the fractions at 200 *μ*g/mL was in the order ethyl acetate > *n* − butanol > residual > *n* − hexane.

Orlistat exhibited an IC_50_ of 10.49 ± 0.83 *μ*g/mL on pancreatic cholesterol esterase activity which was better than ethyl acetate fraction (15.85 ± 0.57 *μ*g/mL), hydroethanolic extract (24.64 ± 2.28 *μ*g/mL), ethanolic extract (28.99 ± 1.98 *μ*g/mL), *n*-butanol fraction (33.45 ± 1.75 *μ*g/mL), aqueous extract (49.06 ± 0.48 *μ*g/mL), *n-*hexane fraction (49.67 ± 1.42 *μ*g/mL), and residual fraction (52.08 ± 0.18 *μ*g/mL) (Table 1).

### 3.3. Effects of *P. glandulosus* Leaves Extracts and Fractions on Lipid Accumulation Inhibition in SW-872 Adipocytes

**Figure 5:**
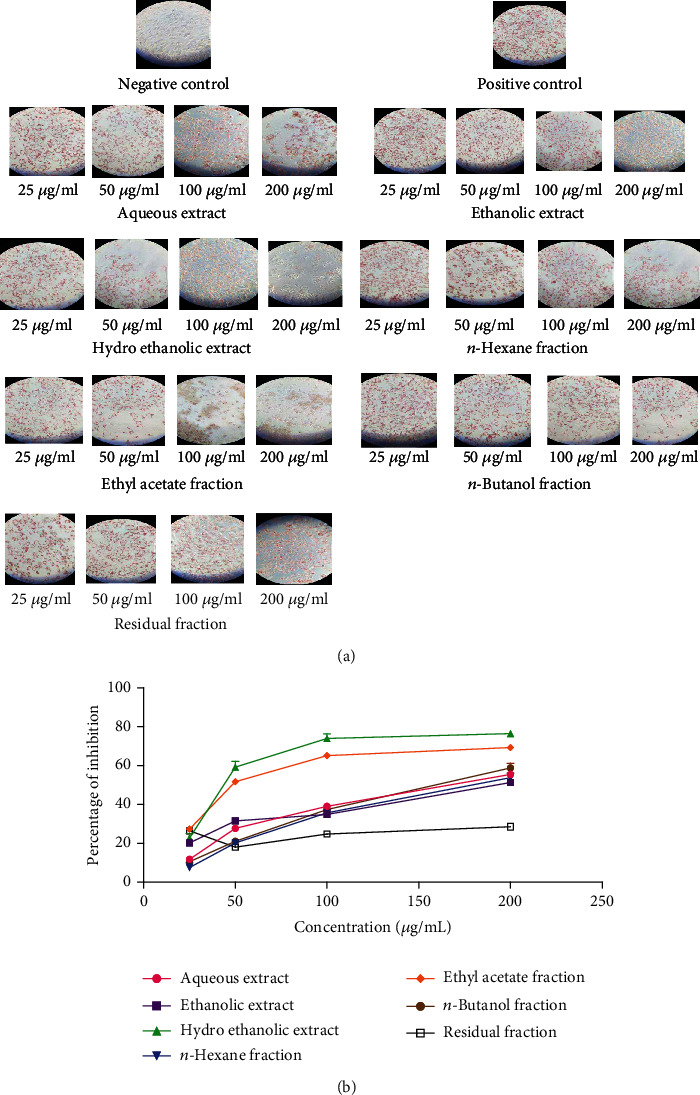
*P. glandulosus* leaves extracts and fractions inhibit lipid accumulation in SW-872 adipocytes. (a) Representative images showing lipid accumulation in cells treated with different concentration of extracts/fractions. (b) Percentage inhibition of lipid accumulation by extracts/fractions. Values are expressed as mean ± SD (*n* = 3); ^∗∗∗^*p* < 0.001 significant differences compared to orlistat.

The results of the Oil Red-O staining are presented in Figure 5(a). The microscopic observation of SW-872 cells showed that the marked red staining in differentiated SW-872 adipocytes indicates the abundant accumulation of lipids (triglycerides), as expected in well-differentiated adipocytes. Cells contained in the positive control wells showed an important accumulation of lipid, while the negative control did not show any lipid accumulation. The addition of different concentrations of *P. glandulosus* leaves extracts and fractions inhibited the lipid accumulation in dose-dependent manner. The greatest inhibitions were observed with the hydroethanolic extract mostly at the concentration 200 *μ*g/mL (76.47%), followed by the ethyl acetate fraction (69.3%) at this same concentration (Figure 5(b)).

The smaller IC_50_ value was obtained with the hydroethanolic extract (60.75 ± 1.04 *μ*g/mL), followed by ethyl acetate fraction (77.12 ± 1.52 *μ*g/mL), *n*-butanol fraction (160.89 ± 4.33 *μ*g/mL), aqueous extract (165.83 ± 1.08 *μ*g/mL), *n-*hexane fraction (175.78 ± 1.52 *μ*g/mL, ethanolic extract (190.24 ± 1.70 *μ*g/mL), and residual fraction (963.04 ± 2.3 *μ*g/mL) (Table 2).

### 3.4. Effects of *P. glandulosus* Leaves Extracts and Fractions on Thrombolytic Activity

Table 3 shows the thrombolytic activity of different extracts and fractions of *P. glandulosus.* It appears that extracts and fractions as well as aspirin showed a dose-dependent response in thrombolytic activity. Aspirin showed better percentages of thrombotic lysis than that of different extracts and fractions at all concentrations. At the concentration 800 *μ*g/mL, hydroethanolic extract showed a lysis thrombotic percentage of 71.43%, followed by ethanolic (57.14%) and aqueous (53.43%) extracts. The lysis thrombotic percentage exhibited by aspirin at this concentration was 89.19%. Among fractions, ethyl acetate exhibited a lysis thrombotic percentage of 82.35%. This percentage was higher than that of *n*-butanol (73.68%), *n-*hexane (54.55%), and residual (50.00%) fractions.

### 3.5. LC- HRMS Analysis Results

**Figure 6:**
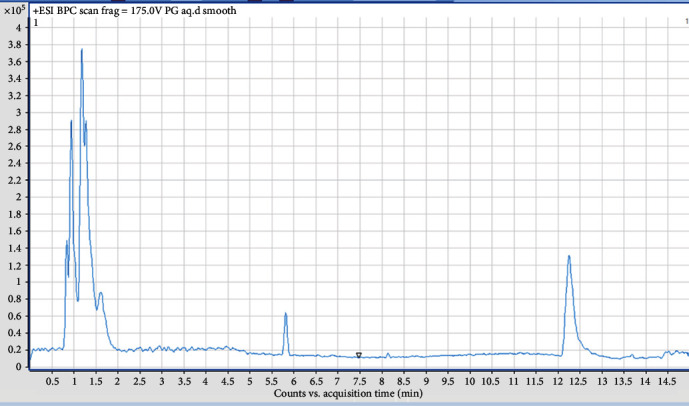
Chromatogram of aqueous leaves extract of *P. glandulosus* showing poor seperation of peaks.

**Figure 7:**
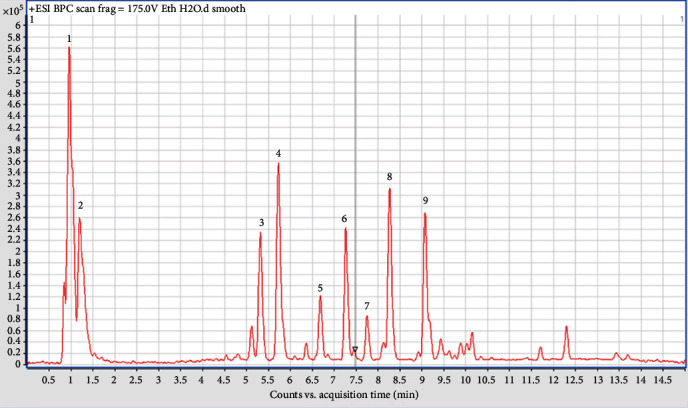
Chromatogram of hydroethanolic (70/30, v/v) leaves extract of *P. glandulosus* showing different peaks. Peaks 3, 7 : 8, and 9 are known compounds.

**Figure 8:**
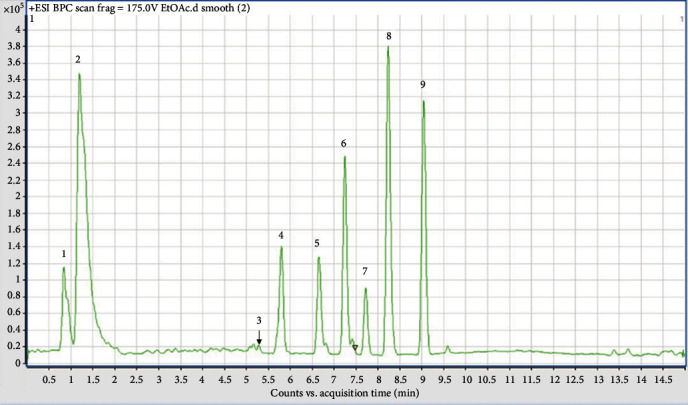
Chromatogram of ethyl acetate leaves fraction of *P. glandulosus* showing different peaks. Peaks 3, 7 : 8, and 9 are known compounds.

The aqueous, hydroethanolic extract, and ethyl acetate fraction of the leaves of *P. glandulosus* were screened by means of LC-HRMS. The chromatogram produced by the aqueous extract did not give a clear picture as to the compounds present (Figure 6). The chromatogram of hydroethanolic extract (Figure 7) and ethyl acetate fraction (Figure 8) was identical with 9 compounds of which some are known compounds while others are unknown. 5-*O-β*-D-glucopyranoside, chrysoeriol 5-*O-β*-D-glucopyranoside, 5,7-dihydroxy-3,2′,4′-trimethoxyflavone, and plectranmicin were revealed as major compounds in both hydroethanolic extract and ethyl acetate fraction. However, hydroethanolic extract presented more compounds than the ethyl acetate fraction that are yet to be identified (Figure 7). The dereplication data of the hydroethanolic extract and ethyl acetate fraction are presented in Tables 4 and 5, respectively. Both hydroethanolic extract presented 4 known compounds and several unknown others.

## 4. Discussion

Atherosclerosis is a multifactorial disease in which dysregulation of lipid metabolism plays a central role from initiation to progression of the atherosclerotic plaque [3, 4]. The rupture of the atherosclerotic plaque is responsible for thrombosis formation which leads to stroke and heart attack major cause of morbidity and mortality over the world [1, 5]. Regulating the disorder of lipid metabolism as well as thrombosis formation is important means to prevent the initiation, progression, and complications of atherosclerosis. In this study, extracts and fractions of *P. glandulosus* leaves were evaluated for their ability to inhibit pancreatic lipase, pancreatic cholesterol esterase, and lipid uptake in SW-872 cells and thrombolytic activity.

Pancreatic lipase plays a role in the breakdown of triglycerides into fatty acids and glycerol [7]. High plasma fatty acids will lead to VLDL production by the liver [8] and their transformation by lipoprotein lipase into smaller atherogenic LDL which have an increased clearance time and able to easily infiltrate the intima [41, 42]. This may be trapped and oxidized by reactive oxygen species, hence the beginning of atherosclerosis. In this study, the inhibitory abilities of extracts and fractions of *P. glandulosus* leaves and orlistat against pancreatic lipase displayed a dose-dependent effect. The hydroethanolic extract (IC_50_, 39.16 ± 0.17 *μ*g/mL) was the most potent among extract, and ethyl acetate exhibited the greatest antilipase activity among fraction (IC_50,_24.20 ± 0.55 *μ*g/mL). However, these activities were lower than that of orlistat (7.53 ± 0.52 *μ*g/mL) the reference drug. The inhibition of pancreatic lipase activity is expected to limit dietary fat absorption, resulting in delayed triglyceride digestion.

Dietary cholesterol consists of both free and esterified cholesterol. Esterified cholesterols are hydrolyzed by pancreatic cholesterol esterase to release free cholesterol in the small intestines [43]. Moreover, it plays an important role in regulating the incorporation of cholesterol into mixed micelles [44] and its transportation into blood plasma. Elevated cholesterol in blood plasma is related to increased LDL levels and has also been associated with significant mechanical endothelial injury and dysfunction, which promote the infiltration and retention of LDL, inflammation, and foam cell formation in the arterial intima, hence the formation of atherosclerosis plaques [45]. The inhibition of pancreatic cholesterol esterase by the extracts and fractions of *P. glandulosus* leaves (hydroethanolic, IC_50,_24.64 ± 2.28 *μ*g/mL and ethyl acetate, IC_50_, 15.85 ± 0.57 *μ*g/mL) may therefore play an important role in reduction of dietary cholesterol absorption although their activities were not comparable to Orlistat (IC_50_ = 10.49 ± 0.83 *μ*g/mL).

Several studies reported that phenolic compounds are involved in lipase and cholesterol esterase inhibition activities [7, 19, 46–48]. Thus, the inhibitory activities of these enzymes in this study might be attributed to the presence of 7-O-methyl luteolin 5-*O-β*-D-glucopyranoside, chrysoeriol 5-*O-β*-D-glucopyranoside, 5,7-dihydroxy-3,2′,4′-trimethoxyflavone, and plectranmicin revealed as major compounds in both hydroethanolic and ethyl acetate extracts through LC-HRMS analysis. The hydroxyl groups present in phenolic compounds are reported to form hydrophobic interactions with amino acid residues of pancreatic lipase [20]. The interaction of phenolic compounds with cholesterol esterase is due to the presence of potent cholesterol esterase inhibitory sites, especially the interaction with the catalytic triad and oxyanion hole residues [49]. Our previous study also reported that hydroethanolic extract and ethyl acetate fraction have antioxidant properties [28, 29], and it is shown that a positive correlation exists between phenolic compounds, antioxidant activity of plant extracts, and digestive enzyme inhibitory activity [50, 51].

Lipid accumulation reflects the process of adipogenesis while the differentiation of preadipocytes involves many stages related to obesity [52]. Today studies have aimed to reduce obesity by focusing on decreasing preadipocyte differentiation and proliferation, inhibiting lipogenesis, and increasing lipolysis. Adipocyte differentiation and accumulation of fat have been associated with the development of obesity [53]. An increase in adipocyte number and mass results from adipocyte differentiation process that generates mature adipocytes from preadipocytes [52]. Chronic consumption of a high fat diet has been shown to increase adipogenesis. Fats are transported to the blood system and delivered to the adipose tissue and liver, leading to lipid accumulation and the development of adipogenesis [54, 55]. In this study, adipogenesis in SW-872 was induced by oleic acid treatment, which is actually a potent adipogenic promoter. In the present study, lipid uptake inhibition was obtained in dose-dependent manner with the different extracts and fractions of *P. glandulosus* leaves. Hydroethanolic extract exhibited the greatest lipid uptake inhibition (IC_50_, 45.42 *μ*g/mL), followed by ethyl acetate fraction (IC_50_, 47.77 *μ*g/mL). These results suggested that *P. glandulosus* led to a strong inhibition of adipogenesis and lipid accumulation under these conditions.

The rupture of an atherosclerotic plaque is associated with thrombotic vessel occlusion which leads to the development of stroke and heart attack, the main complication of atherosclerosis [5]. Thrombin is very important in converting soluble fibrinogen into insoluble fibrin during coagulation cascade. It also activates coagulation factors that trigger mobilization of activated platelets to join the circulation [56]. Thrombin is also proinflammatory enhancing the proliferation and mobilization of smooth muscles cells [57]. Contrariwise, antithrombin decreases platelet activation by encouraging the secretion of prostaglandin from vasculoendothelial cells [58]. Aspirin attenuates thrombin-mediated coagulant and promotes fibrinolysis through acetylation of lysine components in fibrinogen [59]. Thus, aspirin was a perfect reference drug for this study. A couple of studies have reported the antithrombotic activity of plant extracts with improved efficacy for preventing or treating arterial or venous thrombosis [60, 61]. In the present study, we tested the effect of *P. glandulosus* leaves on antithrombotic activity. Contrary to the positive control (aspirin) in the thrombolysis analysis (clot dissociation), the negative control (distilled water) demonstrated that water will never activate clot dissociation. However, in the presence of the different extracts or fractions, important thrombolytic activity was observed in a dose dependent-manner. The greatest percentages of clot dissociation were obtained with aspirin followed by ethyl acetate fraction. Among extracts, a better percentage of clot dissociation was obtained with hydroethanolic extract. At the concentration 800 *μ*g/mL, the percentage of clot dissociation observed with aspirin was 89.19 ± 1.8%. At this same concentration, ethyl acetate exhibited 82.35 ± 1.7%, while hydroethanolic caused clot dissociation with a percentage of 71.43 ± 0.9%. The dissolution of blood clot utilizes the fibrinolytic system which corresponds to the conversion of plasminogen into an active enzyme, plasmin whose principal function is blood clot dissolving [62]. Extract and fraction of *P. glandulosus* leaves would thus have acted by utilizing this fibrinolysis system. Flavonoids present in extracts, and fractions might be responsible for the thrombolytic activity observed in this study [22]. Also, alkaloids, tannins, and saponins, which were identified in the plant in previous studies [28–31], are involved in thrombolytic activities [23].

## 5. Conclusions

The study showed that extracts and fractions of *P. glandulosus* leaves possess in vitro lipase, cholesterol esterase, and lipid uptake inhibitory properties as well as antithrombotic activities. The ethyl acetate fraction and hydroethanolic extract showed the best activities though not comparable to the reference drugs (orlistat for enzyme inhibitions and aspirin for antithrombotic activity) used in this study. Thus, *P. glandulosus* leaves could find importance in the prevention and management of atherosclerosis through inhibition of lipid digestive enzymes, inhibition of lipid uptake, and antithrombotic activity. Phytochemical studies did not reveal the presence of any new compound. However, already known compounds (7-O-methyl luteolin 5-O-ß-D-glucopyranoside, chrysoeriol 5-O-ß-D-glucopyranoside, 5,7-dihydroxy-3,2′,4′-trimethoxyflavone, and plectranmicin) were identified which may be responsible for the shown activities of *P. glandulosus*.

## Figures and Tables

**Table 1:** IC_50_ of extracts and fractions of *P. glandulosus* leaves in lipase and cholesterol esterase activity.

	IC_50_ (*μ*g/mL)	
	Pancreatic lipase	Pancreatic cholesterol esterase
Aqueous extract	79.62 ± 1.26	49.06 ± 0.48^f^
Ethanolic extract	56.62 ± 2.12^e^	28.99 ± 1.98^d^
Hydroethanolic extract	39.16 ± 0.17^c^	24.64 ± 2.28^c^
*n*-Hexane fraction	86.58 ± 0.07	49.67 ± 1.42^f^
Ethyl acetate fraction	24.20 ± 0.55^b^	15.85 ± 0.57^b^
*n*-Butanol fraction	44.09 ± 1.41^d^	33.45 ± 1.75^e^
Residual fraction	71.15 ± 0.60	52.08 ± 0.18
Orlistat	7.53 ± 0.52^a^	10.49 ± 0.83^a^

^a,b,c,d,e,f^Different superscripts indicate significant difference (*p* < 0.001). Data presented are mean ± standard deviation of samples done in triplicates. Means with same superscripts in same column (a, b, c, d, e, f) are not significantly different from each other at *p* > 0.05.

**Table 2:** IC_50_ of extracts and fractions of *P. glandulosus* leaves on lipid accumulation inhibition in SW-872 adipocytes.

IC_50_ (*μ*g/mL)						
Aqueous extract	Ethanolic extract	Hydroethanolic extract	*n-*Hexane fraction	Ethyl acetate fraction	*n*-Butanol fraction	Residual fraction
165.83 ± 1.08^c^	190.24 ± 1.70	60.75 ± 1.04^a^	175.78 ± 1.52	77.12 ± 1.52^b^	160.89 ± 4.33^c^	963.04 ± 2.3

^a,b,c^Different superscripts indicate significant difference (*p* < 0.001). Data presented are mean ± standard deviation of samples done in triplicates. Means with same superscripts (a, b, c) are not significantly different from each other at *p* > 0.05.

**Table 3:** Thrombolysis effects of *P. glandulosus* leaves extracts and fractions.

Percentage of thrombotic lysis (%)								
Con (*μ*g/mL)	AQ-E	ET-E	HE-E	HEX-F	EA-F	*n*-BUT-F	RES-F	Aspirin
800	53.43 ± 1.5	57.14 ± 2	71.43 ± 0.9	54.55 ± 2.5	82.35 ± 1.7	73.68 ± 2.4	50 ± 1.56	89.19 ± 1.8
400	48.27 ± 0.9	51.85 ± 1.3	66.67 ± 2.3	50.00 ± 2.3	78.57 ± 1.32	61.11 ± 3.4	45 ± 3.6	81.48 ± 2.1
200	42.10 ± 2.2	47.06 ± 2	53.33 ± 1.9	36.36 ± 3.8	69.23 ± 3.44	55.56 ± 2.4	41.03 ± 1.9	69.23 ± 3.2
100	37.87 ± 1.2	27.27 ± 3.1	44.44 ± 2.2	22.22 ± 2.1	50.00 ± 1.56	53.85 ± 1.3	33.33 ± 2.13	58.33 ± 1.9
50	27.45 ± 1.3	18.18 ± 0.5	36.36 ± 3.9	16.67 ± 4.5	42.86 ± 1.70	38.46 ± 1.1	18.18 ± 3.09	46.15 ± 3.9

Negative control (distilled water) = 3.03%; *n* = 3; AQ-E: aqueous extract; ET-E: ethanolic extract; HE-E: hydroethanolic extract; Hex-F: *n-*hexane fraction; EA-F: ethyl acetate fraction; *n*-BUT-F: *n*-butanol fraction; RES-F: residual fraction.

**Table 4:** Dereplication results for hydroethanolic (70/30, v/v) leaves fraction of *P. glandulosus*.

No.	*t*_*R*_	Ion [M + H]^+^	Suggested compound
1	0.9-1.1	n.d.	Unknown
2	1.3	n.d.	Unknown
3	5.2-5.3	463.1240	7-*O*-Methyl luteolin 5-*O*-*β*-D-glucopyranoside/chrysoeriol 5-*O*-*β*-D-glucopyranoside^∗^
4	5.7	447.0916	Unknown
5	6.6	287.0548	Unknown
6	7.3-7.4	309.0912	Unknown
7	7.7-7.8	345.0959	5,7-Dihydroxy-3,2′,4′-trimethoxyflavone/pilloin^∗^
8	8.2-8.3	345.0948	5,7-Dihydroxy-3,2′,4′-trimethoxyflavone/pilloin^∗^
9	9.0-9.1	359.1094	Plectranmicin

^∗^The different flavonoids have the same m/z. The assignments were done in comparison with the literature [32, 33].

**Table 5:** Dereplication results for ethyl acetate leaves fraction of *P. glandulosus*.

No.	*t*_*R*_	Ion [M + H]^+^	Suggested compound
1	1.2-1.3	n.d.	Unknown
2	1.8-1.9	n.d.	Unknown
3	5.2-5.3	463.1240	7-*O*-Methyl luteolin 5-*O-β*-D-glucopyranoside/chrysoeriol 5-*O-β*-D-glucopyranoside^∗^
4	5.7	447.0914	Unknown
5	6.6	287.0501	Unknown
6	7.2-7.3	271.0595	Unknown
7	7.6-7.7	345.0948	5,7-Dihydroxy-3,2′,4′-trimethoxyflavone/pilloin^∗^
8	8.2-8.3	345.0948	5,7-Dihydroxy-3,2′,4′-trimethoxyflavone/pilloin^∗^
9	9.0-9.1	359.1060	Plectranmicin

^∗^The different flavonoids have the same m/z. The assignments were done in comparison with the literature [32, 33].

## Data Availability

All data types used are included in the manuscript.

## Abbreviations

ATCC: American Type Culture Collection
BSA: Bovine serum albumin
DMEM/Ham's F-12: Dulbecco's Modified Eagle Medium/Nutrient Mixture F-12
HDL: High-density lipoprotein
IDl: Intermediate density lipoprotein
IC_50_: Inhibitory concentration at 50 percent
IL-6: Interleukine-6
IL-8: Interleukine-8
INF- *γ*: Interferon-*γ*
LDL: Low-density lipoprotein
LC-HRMS: Liquid chromatography-high resolution mass spectrometry
PBS: Phosphate*-*buffer saline
*p-NPB*: *p*-Nitrophenyl butyrate
MCP-1: Chemoattractant protein 1
TNF-*α*: Tumor necrosis factor alpha
VLDL: Very low-density lipoprotein.

## References

[1] World Health Organization (WHO) (2020). *The Top 10 Causes of Death*.

[2] World Health Organisation (WHO) (2021). *Cardiovascular Diseases*.

[3] Ross R. (1999). Atherosclerosis: an inflammatory disease. *The New England Journal of Medicine*.

[4] Hasan S. T., Zingg J.-M., Kwan P., Noble T., Smith D., Meydani M. (2014). Curcumin modulation of high fat diet-induced atherosclerosis and steatohepatosis in LDL receptor deficient mice. *Atherosclerosis*.

[5] Bentzon J. F., Otsuka F., Virmani R., Falk E. (2014). Mechanisms of plaque formation and rupture. *Circulation Research*.

[6] Machhi J. P., Shah N. N. (2012). Study of Antiatherosclerotic activity of polyherbal preparation using rat as an experimental animal model. *International Journal of Pharmaceutical Sciences and Research*.

[7] Birari R. B., Bhutani K. K. (2007). Pancreatic lipase inhibitors from natural sources: unexplored potential. *Drug Discovery Today*.

[8] Klop B., Wouter J. J., Rabelink T. J., Castro C. M. A. (2012). A physician's guide for the management of hypertriglyceridemia: the etiology of hypertriglyceridemia determines treatment strategy. *Panminerva Medica*.

[9] Paththinige S. C., Sirisena N. D., Dissanayake V. H. W. (2017). Genetic determinants of inherited susceptibility to hypercholesterolemia – a comprehensive literature review. *Lipids in Health and Disease*.

[10] Lv J., Yang R., Guo Y., Shi Z. Z., Jinshan Y. (2017). Ox-LDL-induced MicroRNA-155 promotes autophagy in human endothelial cells via repressing the Rheb/mTOR pathway. *Cellular Physiology and Biochemistry*.

[11] Kwaifa I. K., Bahari H., Yong Y. K., Noor S. M. (2020). Endothelial dysfunction in obesity-induced inflammation: molecular mechanisms and clinical implications. *Biomolecules*.

[12] Yanai H., Yoshida H. (2019). Beneficial effects of adiponectin on glucose and lipid metabolism and atherosclerotic progression: mechanisms and perspectives. *International Journal of Molecular Sciences*.

[13] Gustafson B. (2010). Adipose tissue, inflammation and atherosclerosis. *Journal of Atherosclerosis and Thrombosis*.

[14] Naci H., Brugts J., Ades T. (2013). Comparative tolerability and harms of individual statins: a study-level network meta-analysis of 246 955 participants from 135 randomized, controlled trials. *Circulation: Cardiovascular Quality and Outcomes*.

[15] Ballinger A., Peikin S. R. (2002). Orlistat: its current status as an anti-obesity drug. *European Journal of Pharmacology*.

[16] Drew B. S., Dixon A. F., Dixon J. B. (2007). Obesity management: update on orlistat. *Vascular Health and Risk Management*.

[17] Abeysekera W. P. K. M., Arachchige S. P. G., Ratnasooriya W. D. (2017). Bark extracts of Ceylon cinnamon possess antilipidemic activities and bind bile acids In Vitro. *Evidence-Based Complementary and Alternative Medicine*.

[18] Zhang H.-L., Wu Q.-X., Wei X., Qin X.-M. (2020). Pancreatic lipase and cholesterol esterase inhibitory effect of *Camellia nitidissima* chi flower extracts *In Vitro* and *In Vivo*. *Food Bioscience*.

[19] Chatatikun M., Kwanhian W. (2020). Phenolic profile of nipa palm vinegar and evaluation of its antilipidemic activities. *Evidence-Based Complementary and Alternative Medicine*.

[20] Mi S., Liu J., Liu X., Fu Y., Yi J., Cai S. (2021). Inhibitory effects of myricetrin and dihydromyricetin toward *α*-glucosidase and pancreatic lipase with molecular docking analyses and their interaction. *Journal of Food Quality*.

[21] Ejaz A., Wu D., Kwan P., Meydani M. (2009). Curcumin inhibits adipogenesis in 3T3-L1 adipocytes and angiogenesis and obesity in C57/BL mice. *The Journal of Nutrition*.

[22] Guerrero M. S., Torres J. S., Nunez M. J. (2008). Extraction of polyphenols from white distilled grape pomace: optimization and modelling. *Bioresource Technology*.

[23] Shovo M. A., Tona M. R., Mouah J. (2021). Computational and pharmacological studies on the antioxidant, thrombolytic, anti-inflammatory, and analgesic activity of Molineria capitulata. *Current Issues in Molecular Biology*.

[24] Ngassoum M. B., Jirovetz L., Buchbauer G., Fleischhaker W. (2001). Investigation of essential oils ofPlectranthus glandulosusHook f. (Lamiaceae) from Cameroon. *Journal of Essential Oil Research*.

[25] Tatsadjieu N. L., Etoa F.-X., Mbofung C. M. F., Ngassoum M. B. (2008). Effect of Plectranthus glandulosus and Ocimum gratissimum essential oils on growth of Aspergillus flavus and aflatoxin B1 production. *Tropicultura*.

[26] Goudoum A., Tinkeu L. S. N., Ngassoum M. B., Mbofung C. M. (2013). Persistence of active compounds of essential oils of Clausena anisata (rutaceae) and Plectranthus glandulosus (Labiateae) used as insecticides on maize grains and flour. *African Journal of Food, Agriculture., Nutrition and Development*.

[27] Annie L. F. M., Edwige L. N., Calvin B. Z., Hélène-Laure N., Phillipe E. K. B., Alain B. D. (2016). Antinociceptive and anti-inflammatory effects of the aqueous leaves extract of Plectranthus glandulosus. Hook. F. (Lamiaceae) in mice and rats. *Pharmacologia*.

[28] Zouheira D., Wansi S. L., Bouobouo L. P. (2020). Antioxidant and anti-inflammatory activity of Plectranthus glandulosus leaves extracts. *International Journal of Pharmaceutical Sciences Review and Research*.

[29] Zouheira D., Agbor G. A., Singh R., Kamani S. L. P. (2020). In vitro antioxidant properties and inhibitory effect of extracts and fractions of Plectranthus glandulosus leaves on copper sulfate (CuSO4)-induced oxidation in human low-density lipoprotein. *Journal of Drug Delivery & Therapeutics*.

[30] Egwaikhide P. A., Gimba C. E. (2007). Analysis of the phytochemical content and anti-microbial activity of Plectranthus glandulosus whole plant. *Middle-East Journal of Scientific Research*.

[31] Danga Y. S. P., Nukenine E. N., Younoussa L., Esimone C. O. (2014). Phytochemicals and larvicidal activity of Plectranthus glandulosus (lamiaceae) leaves extracts against anopheles gambiae, aedes aegypti and culex quinquefasciatus (diptera: culicidae). *International Journal of Pure and Applied Zoology*.

[32] Tsopmejio J. P., Momeni J., Nkouam Tsopjio F. (2019). Bioactive secondary metabolites from *Plectranthus glandulosus* Hook. (Lamiaceae). *Phytochemistry Letters*.

[33] Nanmeni G., Tedonkeu A. T., Fankam A. G. (2021). An efflux pumps inhibitor significantly improved the antibacterial activity of botanicals from Plectranthus glandulosus towards MDR phenotypes. *The Scientific World Journal*.

[34] Tedonkeu A. T., Tamokou J.-D.-D., Mpetga J. D. S. (2021). A new antimicrobial nor-friedelane-type triterpenoid and other constituents fromPlectranthus glandulosusHook. f. (Lamiaceae). *Natural Product Research*.

[35] Kim Y. S., Lee Y. M., Kim H. (2010). Anti-obesity effect of *Morus bombycis* root extract: anti-lipase activity and lipolytic effect. *Journal of Ethnopharmacology*.

[36] Adisakwattana S., Intrawangso J., Hemrid A., Chanathong B., Mäkynen K. (2012). Extracts of edible plants inhibit pancreatic lipase, cholesterol esterase and cholesterol micellization, and bind bile acids. *Food Technology and Biotechnology*.

[37] Dordevic A. L., Konstantopoulos N., Cameron-Smith D. (2014). 3T3-L1 preadipocytes exhibit heightened monocyte-chemoattractant protein-1 response to acute fatty acid exposure. *PLoS One*.

[38] Ramírez-Zacarías J. L., Castro-Muñozledo F., Kuri-Harcuch W. (1992). Quantitation of adipose conversion and triglycerides by staining intracytoplasmic lipids with Oil Red O. *Histochemistry*.

[39] Jang Y. S., Wang Z., Lee J.-M., Lee J.-Y., Lim S. (2016). Screening of Korean natural products for anti-adipogenesis properties and isolation of kaempferol-3-O-rutinoside as a potent anti-adipogenetic compound from Solidago virgaurea. *Molecules*.

[40] Prasad S., Kashyap R. S., Deopujari J. Y., Purohit H. J., Taori G. M., Daginawala H. F. (2007). Effect of Fagonia arabica (Dhamasa) on in vitro thrombolysis. *BMC Complementary and Alternative Medicine*.

[41] Capell W. H., Zambon A., Austin M. A., Brunzell J. D., Hokanson J. E. (1996). Compositional differences of LDL particles in normal subjects with LDL subclass phenotype A and LDL subclass phenotype B. *Arteriosclerosis, Thrombosis, and Vascular Biology*.

[42] Björnheden T., Babyi A., Bondjers G., Wiklund O. (1996). Accumulation of lipoprotein fractions and subfractions in the arterial wall, determined in an In Vitro perfusion system. *Atherosclerosis*.

[43] Kumar P., Umamaheswari T. S. M., Jagannath V. S. P. (2011). Cholesterol esterase enzyme inhibitory and antioxidant activities of leaves of Camellia sinensis (L.) Kuntze. Using in vitro models. *International Journal of Pharmaceutical Sciences and Research*.

[44] Myers-Payne S. C., Hui D. Y., Brockman H. L., Schroeder F. (1995). Cholesterol esterase: a cholesterol transfer protein. *Biochemistry*.

[45] Tjaden K., Pardali E., Waltenberger J. (2015). Hypercholesterolemia induces vascular cell dysfunction: molecular basis for atherosclerosis. *Austin Journal of Vascular Medicine*.

[46] Trautwein E. A., Duchateau G. S. M. J. E., Lin Y., Mel'nikov S. M., Molhuizen H. O. F., Ntanios F. Y. (2003). Proposed mechanisms of cholesterol-lowering action of plant sterols. *European Journal of Lipid Science and Technology*.

[47] Ong S. L., Mah S. H., Lai H. Y. (2016). Porcine pancreatic lipase inhibitory agent isolated from medicinal herb and inhibition kinetics of extracts from Eleusine indica (L.) Gaertner. *Journal of Pharmaceutics*.

[48] Martinez-Gonzalez A. I., Alvarez-Parrilla E., Díaz-Sánchez A. G. (2017). *In vitro* inhibition of pancreatic lipase by polyphenols: a kinetic, fluorescence spectroscopy and molecular docking study. *Food Technology and Biotechnology*.

[49] Sathishkumar T., Suriyakala V., Lakshmanabharathy M. (2020). In vitro cholesterol esterase inhibitory activity of some purified phenolic acids from Agaricus bisporus: an investigation of cardioprotective properties. *Jundishapur Journal of Natural Pharmaceutical Products*.

[50] Wu D.-T., Nie X.-R., Shen D.-D. (2020). Phenolic compounds, antioxidant activities, and inhibitory effects on digestive enzymes of different cultivars of okra (Abelmoschus esculentus). *Molecules*.

[51] Pereira E. D. S. (2021). Araçá (Psidium cattleianum Sabine): bioactive compounds, Antioxidant Activity and Pancreatic Lipase Inhibition. *Ciência Rural*.

[52] Lefterova M. I., Lazar M. A. (2009). New developments in adipogenesis. *Trends in Endocrinology & Metabolism*.

[53] Otto T. C., Lane M. D. (2005). Adipose development: from stem cell to adipocyte. *Critical Reviews in Biochemistry and Molecular Biology*.

[54] Schrauwen P., Westerterp K. R. (2000). The role of high-fat diets and physical activity in the regulation of body weight. *The British Journal of Nutrition*.

[55] Od-Ek P., Deenin W., Malakul W., Phoungpetchara I., Tunsophon S. (2020). Anti-obesity effect of *Carica papaya* in high-fat diet fed rats. *Biomedical Reports*.

[56] Dickneite G., Seiffe D., Diehl K. H., Rogers M. (1995). Pharmacological characterization of a new 4-amidinophenyl-alanine thrombin- inhibitor (CRC 220). *Thrombosis Research*.

[57] Borissoff J. I., Spronk H. M., Ten-Cate H. (2011). The hemostatic system as a modulator of atherosclerosis. *The New England Journal of Medicine*.

[58] Mizutani A., Okajima K., Uchiba M. (2003). Antithrombin reduces ischemia/reperfusion-induced renal injury in rats by inhibiting leukocyte activation through promotion of prostacyclin production. *Blood*.

[59] Undas A., Brummel-Ziedins K. E., Mann K. G. (2007). Antithrombotic properties of aspirin and resistance to aspirin: beyond strictly antiplatelet actions. *Blood*.

[60] Klafke J. Z., da Silva M. A., Rossato M. F. (2012). Antiplatelet, antithrombotic, and fibrinolytic activities of Campomanesia xanthocarpa. *Evidence-Based Complementary and Alternative Medicine*.

[61] Mahmud S., Akhter S., Rahman M. A. (2015). Antithrombotic effects of five organic extracts of Bangladeshi plants in vitro and mechanisms in in silico models. *Evidence-Based Complementary and Alternative Medicine*.

[62] Hossain I., Sakib M. D. H., Mahmood A. A. (2015). Study on in-vitro thrombolytic activity of methanolic extract of Mesua ferrea leaves. *International Journal of Medical and Health Research*.

